# Clinical Efficacy Observation of Acupuncture Treatment for Nonarteritic Anterior Ischemic Optic Neuropathy

**DOI:** 10.1155/2015/713218

**Published:** 2015-05-24

**Authors:** Yali Qin, Wei Yuan, Hui Deng, Zhanmei Xiang, Chao Yang, Xinyun Kou, Shufei Yang, Zhijun Wang, Ming Jin

**Affiliations:** ^1^Department of Ophthalmology, China-Japan Friendship Hospital, No. 2, East Yinghua Street, Chaoyang, Beijing 100029, China; ^2^Beijing University of Chinese Medicine, Beijing 100029, China

## Abstract

*Objective*. To determine whether acupuncture treatment impacts the clinical efficacy of degenerative damage of the optic nerve caused by nonarteritic anterior ischemic optic neuropathy (NAION). *Methods*. 69 patients (93 eyes) with NAION who had been treated by acupuncture which is performed on different acupoints related to eyes by vertical insertion or Fingernail-pressure needle insertion. The best corrected visual acuity, mean defect (MD) and mean light sensitivity (MS) of the visual field, and latency and amplitude of pattern visual evoked potential (P-VEP) were compared before and after treatment. *Results*. After 2, 4, and 8 weeks of treatment, the total effective rates of visual acuity improvement were 74.19%, 78.89%, and 81.71%, respectively, and the decreased MD and increased MS were both statistically significant (*P* < 0.01). When compared with the situation before treatment, the average latency of the P_100_ wave was significantly reduced (*P* < 0.05), and the average amplitude was improved with no statistically significant difference (*P* > 0.05). *Conclusions*. Acupuncture treatment could obviously improve the visual function of patients with NAION and be used as complementary and alternative therapy in clinic.

## 1. Background

Nonarteritic anterior ischemic optic neuropathy is one of the most common acute optic neuropathies in patients over 50 years of age, with an estimated annual incidence of 2.3–10.2 per 100 000 population in the 1990s and 1 in 16 000 subjects in adult Chinese [1–3]. NAION cases are usually related to some risk factors such as diabetes and hypertension [4, 5]. Patients with NAION often suffer from acute, painless unilateral loss of eyesight with a characteristic visual field defect and a hyperaemic, swelling, or pale optic disc [6]. At present, therapies for NAION cover glucocorticoids, drugs that impact the autonomic regulation pathways, neurotrophic drugs, ocular hypotensive agents, antivascular endothelial growth factor (VEGF), hyperbaric oxygen therapy, optic nerve sheath decompression, and others [7, 8]. After several months of treatment, the hyperaemic and swelling optic disc, visual acuity, and visual field can be improved partly. However, some patients are often left with local or entire optic atrophy in the later stages and seriously affected visual functions [9].

A large control study showed that NAION eyes treated during the acute phase with systemic corticosteroids resulted in a significant improvement in visual acuity (69.8%) and visual field (40.1%). Both visual acuity and visual fields kept improving up to about 6 months from onset of NAION and very little thereafter [10]. There is a close relation between the visual field defects and the damage of retinal nerve fiber layer (RNFL) and retinal ganglion cells (RGCs) [11, 12]. But high-dose systemic corticosteroids did not show any beneficial effect in RNFL loss of NAION [13]. There is no consistently targeted and effective therapy for degenerative damage of the optic nerve so far. Acupuncture is one of the most frequently used external treatment methods in traditional Chinese medicine (TCM) and is widely used as an important complementary and alternative medicine (CAM) of mainstream medicine in many countries [14]. So we had carried out a trial to observe the clinical efficacy of acupuncture in the treatment of degenerative damage of the optic nerve caused by NAION.

## 2. Materials and Methods

### 2.1. Subjects

This study conformed with the Code of Ethics of the World Medical Association and the standards established by the Institutional Review Board of the hospital. From December 2011 to December 2014, 69 patients (93 eyes) with NAION had participated in the study. All subjects met with the inclusion and exclusion criteria of the study. All experimental procedures were explained to the subjects, and signed informed consent was obtained prior to participation in the study. Among the patients were 47 males (64 eyes) and 22 females (29 eyes). 45 patients had unilateral NAION and 24 had bilateral NAION of which 9 cases occurred at the same time. The minimum age was 39, the maximum age was 79, and the average was 54.86 ± 14.98 years old. The period before receiving acupuncture treatment ranged from 3 months to 5 years and the average period was 12.68 ± 11.82 months.

### 2.2. Criteria for Diagnostic, Inclusion, and Exclusion

#### 2.2.1. Diagnostic Criteria

According to the NAION diagnostic criteria issued by the traditional Chinese and western medicine diagnosis and treatment of optic nerve diseases in 2007 [15], the following are characteristics of NAION: (1) acute visual loss, usually not accompanied with eye movement pain. (2) The pupil of the affected eye could have relative afferent pupillary defect (RAPD). (3) The optic disc appeared hyperaemic, swollen, or pale. (4) The visual field defects presented with fan or quadrant patterns related to optic nerve damage. (5) The conductive function of the optic nerve could be blocked in the P_100_ wave of the pattern in the visual evoked potential (P-VEP). (6) The retina fluorescein angiography could have evidence of filling delay, defect, or leakage around the optic disc.

#### 2.2.2. Inclusion Criteria

The inclusion criteria were (1) meeting the diagnostic criteria of NAION, (2) being treated by normative medication at least 3 months and being left with local or entire optic atrophy before acupuncture, and (3) an age between 18 and 80 years.

#### 2.2.3. Exclusion Criteria

The exclusion criteria were (1) taking part in other clinical trials and being treated by acupuncture for other diseases, (2) other serious eye diseases that affect visual function, (3) serious organic diseases, such as cancer, heart failure, and hemophilia, (4) mental disorders, (5) pregnant women, and (6) patients with a low compliance in treatment or examination.

### 2.3. Acupuncture Treatments

All acupuncture operations were done by ophthalmologists. Sterile, disposable acupuncture needles (0.25 mm in diameter and 40 mm in length) were used, which were purchased from HuaCheng acupuncture instrument (Suzhou, Jiangsu, China). The used acupoints were BL1 (*Jingming*), EX-HN7 (*Qiuhou*) and GB14 (*Yangbai*) of affected eye, DU19 (*Baihui*), EX-HN1 (*Sishencong*) and bilateral EX-HN6 (*Taiyang*), GB20 (*Fengchi*),* Pizhixue*, LI4 (*Hegu*), and SJ5 (*Waiguan*) [16].

After sterilizing, the acupoints around the eyes (BL1, EX-HN7, and GB14) were inserted by Fingernail-pressure needle insertion (press beside the acupoint with the nail of the thumb or the index finger of the left hand and hold the needle with the right hand, keeping the needle tip closely to the nail, and then insert the acupoint). The needle was inserted about 1 cm; the needle was pointed towards the orbital apex and was continued to be inserted about 0.5–1 cm. Other acupoints were inserted vertically or by Fingernail-pressure needle insertion for 1-2 cm. A dull needling sensation (called* de qi* in TCM) which includes sour, numb, heavy, and aching feelings was observed after the insertion of the needles. An electroacupuncture instrument (SDZ-II), Hua Tuo, Suzhou, Jiangsu, China, was connected with the acupuncture needles of Hegu and Waiguan. The stimulation consisted of disperse-dense waves and the electric current varied from 0.1 mA to 1.0 mA until the patients felt comfortable. The needles were retained for 20 minutes per day. The treatment course was 8 weeks: five times a week.

### 2.4. Outcome Measures

The outcomes were measured by (1) the best corrected visual acuity (standard visual acuity chart), (2) the mean defect and mean light sensitivity of the visual field (OCTOPUS), (3) and the latency and amplitude of the P_100_ wave of P-VEP (ROLAND CONSULT RETI-Port). The best corrected visual acuity, mean defect, and mean light sensitivity of visual field were compared between, before, and after 2, 4, and 8 weeks of treatment with acupuncture. The latency and amplitude of the P-VEP were compared between, before, and after 8 weeks of treatment.

### 2.5. Effective Criteria of Visual Acuity

When the visual acuity was above or equal to 0.1, it was evaluated by improved lines of visual acuity chart. When the visual acuity was below 0.1, it has been graded into blackout, light sense, hand movement, finger movement, 0.02, 0.04, 0.06, and 0.08. According to the effective criteria of visual acuity issued by the Chinese ophthalmology in 2005 [17], effective means being greater than or equal to 4 lines or grades or recovering the level before illness; improved means being greater than or equal to 2 lines or grades; invalid means unchanged or declined visual acuity.

### 2.6. Statistical Analysis

SPSS 20.0 software was used to analyze the visual field and P-VEP. All data were processed by a* t*-test and a nonparametric test and were provided as mean ± standard deviation ($\overset{-}{x}\;\pm \;SD$). The test level of statistical significance was set at an *α* = 0.05.

## 3. Results

### 3.1. Visual Acuity

The distribution of visual acuity before acupuncture treatment is shown in Table 1. After 2 weeks of treatment, the total cases were 69 patients (93 eyes), in which the number of effective eyes was 20, improved 49, and invalid 24. After 4 weeks of treatment, the total cases were 67 patients (90 eyes), the number of effective eyes was 24, improved 47, and invalid 19. After 8 weeks of treatment, the total number of cases was 61 patients (82 eyes), in which the number of effective eyes was 27, improved 40, and invalid 15. Compared with the before treatment status, the total effective rates of visual acuity improvement after 2, 4, and 8 weeks were 74.19% (69 eyes), 78.89% (71 eyes), and 81.71% (67 eyes), respectively (Table 2).

### 3.2. MD (dB) and MS (dB) of Visual Field

**Figure 1:**
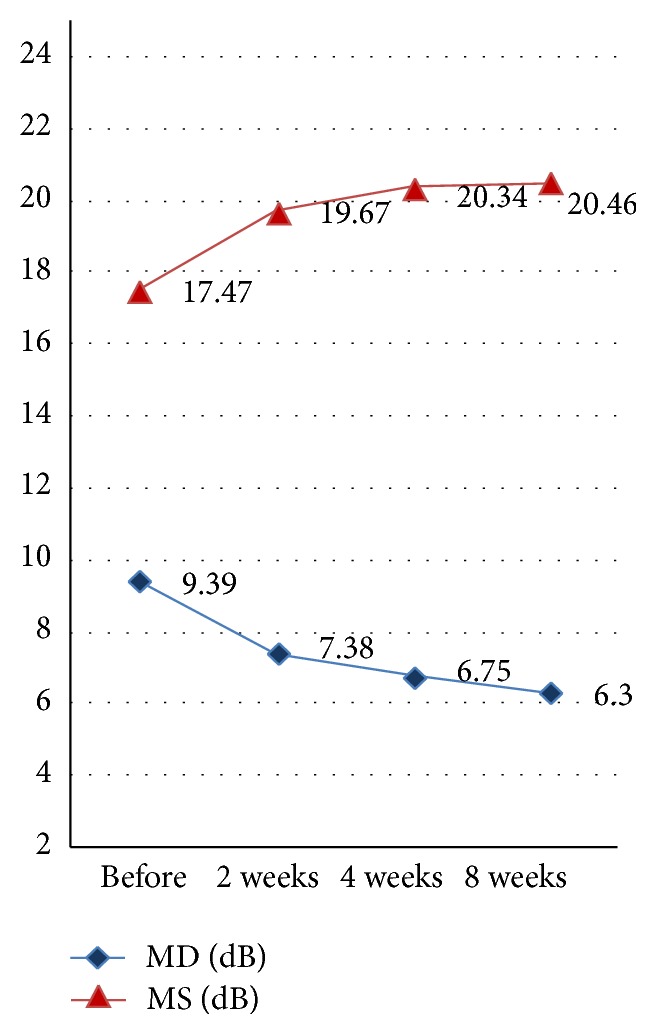
Mean values of MD and MS before and after 2, 4, and 8 weeks of acupuncture treatment.

**Figure 2:**
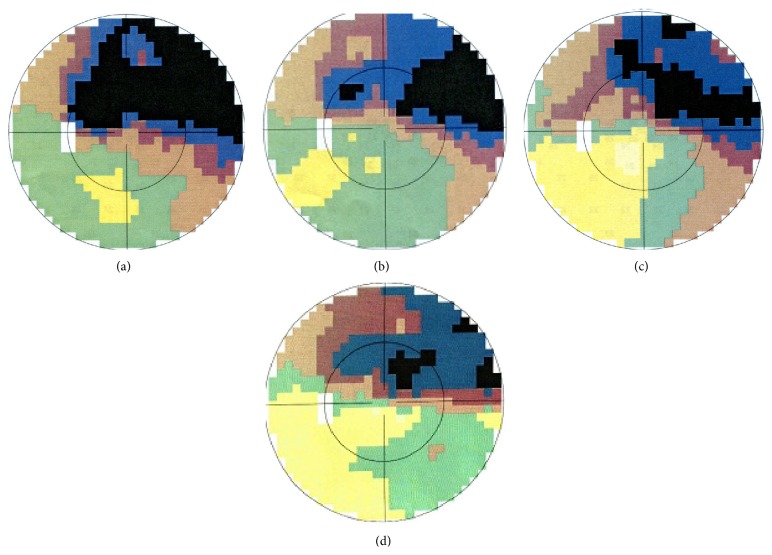
One example of visual field changes with NAION. (a) Before treatment. (b) 2 weeks after treatment. (c) 4 weeks after treatment. (d) 8 weeks after treatment.

The mean defect and mean light sensitivity of the visual field before acupuncture treatment were 9.39 and 17.47 (dB), respectively. After 2, 4, and 8 weeks of acupuncture treatment, the MD was decreased to 7.38, 6.75, and 6.30 (dB), respectively. The MS was increased to 19.67, 20.34, and 20.46 (dB), respectively (Figure 1). The comparative analysis showed that both changes were statistically significant (*P* < 0.01) (Table 3). One example of visual field changes with NAION (Figure 2).

### 3.3. Latency (ms) and Amplitude (*μ*V) of P_100_ Wave of P-VEP

**Figure 3:**
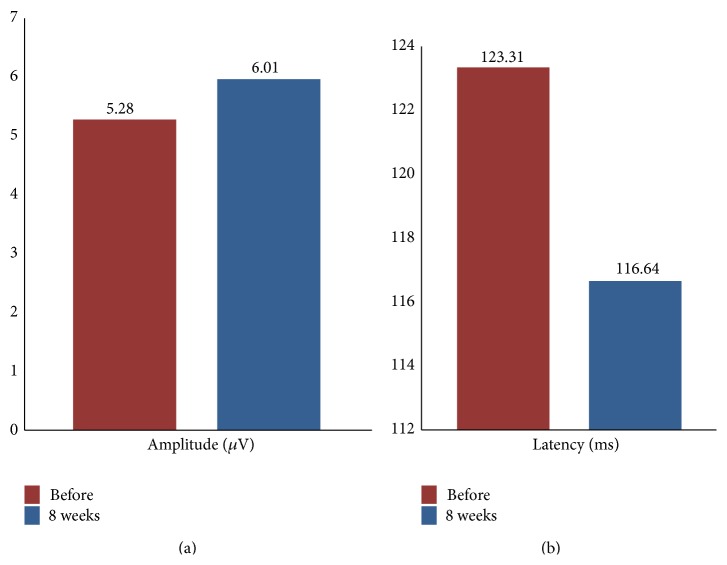
Changes of amplitude and latency before and after 8 weeks of acupuncture treatment.

As shown in Table 4, the average latency of the P_100_ wave before and after 8 weeks of acupuncture treatment was 123.31 and 116.64 (ms), respectively (Figure 3(a)), which was significantly reduced (*P* < 0.05) (Table 4). The average amplitude before and after 8 weeks of acupuncture treatment was 5.28 and 6.01 (*μ*V), respectively (Figure 3(b)), which was improved but with no statistical significant difference (*P* > 0.05) (Table 4).

## 4. Discussion

Acupuncture as one of the most frequently external treatment methods in traditional Chinese medicine has been widely used to treat many neurological diseases or chronic diseases. Treatment with standard acupuncture was well safe and tolerated. During the acupuncture treatments, patients had no abnormal reaction such as dizziness, palpitations, or severe pain. Modern research about acupuncture medicine indicates that acupuncture seems to achieve therapeutic effect through some special ways [18, 19]. For instance, by improving the nerve excitability and expanding peripheral arterioles, acupuncture treatment can promote the conductive function of nerve cells and improve the blood supply to tissue and metabolism. According to the total concept of TCM, the eyes and internal organs are connected with each other through special pathways called meridians and collaterals. Acupuncture could be performed on acupoints of different meridians related to the eyes.

The acupoints around the eyes (BL1, EX-HN7, and GB14) were usually called “eye three points” and commonly used in treatment of eye diseases. Acupuncture of GB20 (Fengchi) could improve the elasticity of blood vessels in the brain such as vertebral-basilar artery and then promote blood circulation of the brain [20]. The study on acupuncture of DU19 (Baihui) and bilateral EX-HN6 (Taiyang) of cerebral ischemia injury rats found that acupuncture treatment can recover the organizational structure of CA3 of hippocampus, decrease the expression of Endothelin- (ET-) 1 and intercellular adhesion molecule- (ICAM-) 1, and increase the expression of factor VIII related antigen (F VIIIR-Ag). Then acupuncture will reduce cerebral ischemia injury and promote the function of vascular endothelial cellular and neurotrophy [21]. Bilateral LI4 (*Hegu*) and SJ5 (*Waiguan*) could be connected with electroacupuncture instrument for enhancing therapeutic effect. Acupuncture, which is performed on acupoints around ocular region and is related to eyes, could play a role in increasing the blood flow velocity and lowering the vascular resistance of carotid artery, ophthalmic artery [19], and short posterior ciliary artery (PCAs) [22] and then improve the blood circulation and blood perfusion of arteries around optic disc.

*Pizhixue*, also called “Qiaoming,” is corresponding to the area of occipital lobe and visual cortex. We have realized from a study that injured occipital cortex will result in the damage of the lateral geniculate nucleus and optic tract and the loss of retinal ganglion cells [23]. Structural and functional reorganization of occipital regions are present in an individual with a longstanding history of severe visual impairment [24]. A clinical observation found that adding Qiaoming to the common acupoints in treating optic atrophy could improve the curative effect (*P* < 0.05) [25]. It was speculated that acupuncture could promote the recovery of function and construction of visual cortex, improve the conduction of neural signals, and prevent degeneration of RGCs and axon.

To our knowledge, this is the first report about acupuncture treatment for degenerative damage of the optic nerve caused by NAION. In our study, after 2, 4, and 8 weeks of acupuncture treatment, the visual acuity and mean light sensitivity of the visual field were both obviously improved. The mean defect of the visual field and average latency of the P-VEP were reduced gradually. It suggests that regular and continuous acupuncture treatment contributed to the recovery of the visual function of these patients. This may be because acupuncture promoted the blood circulation of artery in the brain and eyes and around the optic disc. Meanwhile, it maybe increased the excitability of optic nerve cells which were damaged in NAION. Finally, the acupuncture repaired and reconstructed the visual pathways.

Although the results indicate an improvement of visual function after acupuncture treatment, there is no sufficient evidence to support the functional mechanism of preventing the aggravation of optic atrophy. Besides, due to the lack of funds and sample, we just carried out a before and after self-controlled study and did not design a randomized control group. This is a great regret. In order to further explore the functional mechanism of acupuncture treatment and provide a new therapy for NAION, we plan to carry out a more integrative study which contains more patients with NAION and more reasonable designs in the future.

## Figures and Tables

**Table 1:** Distribution of visual acuity before acupuncture treatment.

Visual acuity	Number of eyes
≥1.0	9
0.6–<1.0	17
0.4–<0.6	22
0.2–<0.4	21
0.1–<0.2	15
<0.1	9
Total	93

**Table 2:** Improvement of visual acuity after 2, 4, and 8 weeks of acupuncture treatment.

After treatment	Total eyes	Effective eyes	Improved eyes	Invalid eyes	Total effective rates
2 weeks	93	20	49	24	74.19% (69 eyes)
4 weeks	90	24	47	19	78.89% (71 eyes)
8 weeks	82	27	40	15	81.71% (67 eyes)

**Table 3:** MD and MS of visual field ($\overset{-}{x}\pm \text{SD}$).

Groups	MD (dB)	MS (dB)
Before treatment	9.39 ± 6.12	17.47 ± 6.28
2 weeks	7.38 ± 6.10^**∗**^	19.67 ± 6.43^**∗**^
4 weeks	6.75 ± 5.96^**∗**^	20.34 ± 6.26^**∗**^
8 weeks	6.30 ± 5.89^**∗**^	20.46 ± 6.46^**∗**^

^**∗**^ *P* < 0.01.

**Table 4:** Latency and amplitude ($\overset{-}{x}\pm \text{SD}$).

Groups	Latency (ms)	Amplitude (*μ*V)
Before treatment	123.31 ± 17.43	5.28 ± 2.95
8 weeks	116.64 ± 15.55^∗∗^	6.01 ± 2.63^∗∗∗^

^∗∗^ *P* < 0.05, ^∗∗∗^ *P* > 0.05.
